# Serological and molecular detection of *Neospora caninum* and *Toxoplasma gondii* in human umbilical cord blood and placental tissue samples

**DOI:** 10.1038/s41598-020-65991-1

**Published:** 2020-06-03

**Authors:** Pâmella Oliveira Duarte, Leandra Marla Oshiro, Namor Pinheiro Zimmermann, Bárbara Guimarães Csordas, Doroty Mesquita Dourado, Jacqueline Cavalcante Barros, Renato Andreotti

**Affiliations:** 1 Programa de Pós-Graduação em Doenças Infecciosas e Parasitárias- Universidade Federal de Mato Grosso do Sul (UFMS), Campo Grande, MS Brasil; 2 Laboratório de Biologia Molecular do Carrapato, Departamento de Sanidade Animal, Embrapa Gado de Corte, Campo Grande, MS Brasil; 3 Professor do Curso de Medicina Veterinária do Centro Universitário da Grande Dourados-UNIGRAN, Dourados, Brasil; 4 Bolsista de Pós-Doutorado- FUNDAPAM, Laboratório de Biologia Molecular do Carrapato, Departamento de Sanidade Animal, Embrapa Gado de Corte, Campo Grande, MS Brasil; 5 Laboratório de Toxonologia e Plantas Medicinais-Uniderp Agrárias, Campo Grande, Brasil; 6 Empresa Brasileira de Pesquisa Agropecuária, Embrapa Gado de Corte, Campo Grande, MS Brasil

**Keywords:** Molecular biology, Diseases

## Abstract

Neosporosis primarily affects cattle and dogs and is not currently considered a zoonotic disease. Toxoplasmosis is a zoonosis with a worldwide distribution that is asymptomatic in most cases, but when acquired during pregnancy, it can have serious consequences. The seropositivity rates determined by the indirect fluorescent antibody test for *Neospora caninum* (*N. caninum*) and *Toxoplasma gondii* (*T. gondii*) were 24.3% (49 samples) and 26.8% (54 samples), respectively. PCR positivity for *N. caninum* was observed in two samples of cord blood (1%) using the Nc5 and ITS1 gene, positivity for *T. gondii* was observed in 16 samples using the primer for the B1 gene (5.5% positivity in cord blood and 2.5% positivity in placental tissue). None of the samples showed structures characteristic of tissue cysts or inflammatory infiltrate on histopathology. Significant associations were observed only between *N. caninum* seropositivity and the presence of domestic animals (p = 0.039) and presence of dogs (p = 0.038) and between *T. gondii* seropositivity and basic sanitation (p = 0.04). This study obtained important findings regarding the seroprevalence and molecular detection of *N. caninum* and *T. gondii* in pregnant women; however, more studies are necessary to establish a correlation between risk factors and infection.

## Introduction

*Neospora caninum* is an obligate intracellular parasite belonging to the phylum Apicomplexa and was first identified in 1984 in the central nervous system and skeletal muscle of dogs in Norway^1^. *N. caninum* has a wide range of hosts^2,3^, but neosporosis is a disease that primarily affects cattle and dogs, and canids are definitive hosts. The forms of infection are essentially the same as those of toxoplasmosis, occurring horizontally in herbivores via intake of water or foods contaminated by oocysts and in carnivores via ingestion of tissues infected with tachyzoites or tissue cysts. Vertical transmission may also occur, and *N. caninum* is very efficiently transplacentally transmitted in cattle, which may cause abortion^2^ or birth of infected and asymptomatic calves^2,3^.

*Toxoplasma gondii* can infect all warm-blooded animals, including mammals, birds, and humans^4^. Toxoplasmosis is an infection caused by the parasite *T. gondii* and may be congenital or acquired^5^. Intake of oocysts present in the environment and consumption of undercooked meat infected with tissue cysts are the two main forms of transmission in acquired infection^5,6^. Congenital transmission occurs after primary infection during pregnancy^7^.

The infection in most cases is asymptomatic, the mother develops temporary parasitemia. However, focal lesions can develop in the placenta, and the fetus may be infected. Slightly diminished vision is characteristic of mild disease, whereas severely all children may present with retinochoroiditis, hydrocephalus, seizures and intracerebral calcification^8^.

The diseases caused by *T. gondii* and *N. caninum* have similar characteristics, such as neurological conditions and reproductive pathologies, due to the morphological, genetic and immunological similarities of the two parasites^9,10^.

The pathological, immunological and epidemiological aspects of neosporosis in human pregnancies are still unknown, since viable *N. caninum* has not been isolated from human tissues so far. However, knowing that this parasite has a wide range of intermediate hosts^2,3^, the possibility of human infection should not be ruled out. If there is a possibility of vertical transmission in humans, we believe that the evolution and severity of the infection is dependent on the mother’s gestational age and the virulence of the strain causing the infection, as occurs in other animal species^11,12^.

Anti-*N. caninum* antibodies have been reported in humans in several studies^13–16^, and its zoonotic potential is still uncertain. Studies conducted with human placental tissue and umbilical cord blood for detecting *N. caninum* remain scarce in the literature. Therefore, the objective of this study was the molecular and serological detection of *N. caninum* and/or *T. gondii* in blood samples from the umbilical cords and placental tissues of pregnant women.

## Results

The pregnant women who participated in the study had a mean age of 27.5 ± 6.022 years and were at a gestational age of 39 ± 1.4 weeks, and 13.9% of the women did not have appropriate prenatal care (28/201) as recommended by the competent organs of Brazil, which recommends six or more prenatal visits.

### Serology

Of the 201 samples analyzed, 24.3% were positive for IgG anti-*N. caninum* antibodies (Table 1), and no sample was positive for IgM antibodies. For *T. gondii*, 26.8% of the samples were positive for the presence of IgG antibodies (Table 2), and no sample was positive for IgM antibodies. Of all samples analyzed, 8.4% presented seropositivity for both parasites. Western blot positive samples corroborate IFAT results, showing reactivity with 29 kDa protein (Supplementary Fig. S1). Positive samples for *N. caninum* in PCR, despite not being positive by IFAT, were western blot positive.

**Table 1:** IFAT for IgG anti-*N. caninum* antibodies in cord serum.

Neospora caninum			
Number of samples	IFAT (1:50)	IFAT (1:100)	IFAT (1:200)
	+/%	+/%	+/%
201	49/24.3	9/4.4	3/1.4

**Table 2:** IFAT for IgG anti*-T. gondii* antibodies in cord serum.

Toxoplasma gondii					
Number of samples	IFAT (1:64)	IFAT (1:128)	IFAT (1:256)	IFAT (1:512)	IFAT (1:1024)
	+/%	+/%	+/%	+/%	+/%
201	54/26.8	29/14.4	9/4.4	6/2.9	4/1.9

Statistical analyses of the *N. caninum* data showed significant associations (p < 0.05) between seropositivity and the presence of domestic animals and the presence of dogs. For *T. gondii*, a significant association (p < 0.05) between seropositivity and basic sanitation (Table 3) was observed.

**Table 3:** Seroprevalence of IgG antibodies against *N. caninum* and *T. gondii* associated with risk factors for infection.

*Neospora caninum*				
Parameters	N (%)		p value	Odds ratio (CI 95%)
	IgG positive	IgG negative		
**Age**				
≤30	27/132 (55.1%)	105/132 (69.1%)	0.102	
31–40	20/65 (40.8%)	45/65 (29.6%)		—
>40	2/4 (4.1%)	2/4 (1.3%)		
**Total**	**49**/**201**	**152**/**201**		
Consumption of raw/undercooked meat	11/49 (22%)	38/49 (25%)	0.849	0.868 (0.404–1.866)
Work or leisure activities involving soil	5/21 (10)	16/21 (10.5)	0.537	0.896 (0.312–2.569)
Domestic animals	38/130 (77.5)	92/130 (60.5)	0.039*	2.253 (1.069–4.749)
Cat	8/36 (16)	28/36 (18)	0.833	0.864 (0.365–2.045)
Dog	35/120 (71)	85/120 (56)	0.038*	1.971 (0.981–3.959)
Basic sanitation	17/65 (35)	48/65 (31.5)	0.727	0.869 (0.440–1.716)
***Toxoplasma gondii***				
	IgG positive	IgG negative		
**Age**				
≤30	35/132 (64.8%)	97/132 (66%)	0.421	
31–40	19/65 (35.2%)	46/65 (31.3%)		—
>40	0/4 (0%)	4/4 (2.7%)		
**Total**	**54**/**201**	**147**/**201**		
Consumption of raw/undercooked meat	41/77 (76%)	36/77 (24.5%)	0.555	0.978 (0.472–2.025)
Work or leisure activities involving soil	8/21 (15)	13/21 (9)	0.307	1.698 (0.665–4.290)
Domestic animals	17/110 (31)	93/110 (63)	0.511	1.224 (0.650–2.457)
Cat	8/36 (15)	28/36 (19)	0.541	0.739 (0.314–1.740)
Dog	37/120 (68.5)	83/120 (56.5)	0.145	1.678 (0.867–3.248)
Basic sanitation	30/136 (55.5)	106/136 (72)	0.04*	0.483 (0.253–0.923)

### Molecular biology

Of the 201 umbilical cord blood samples analyzed, two samples (1%) were Nc5 PCR-positive for *N. caninum* and these same samples were positive for ITS1 (GenBank: MN731361), but no sample of the placenta was positive.

**Figure 1:**
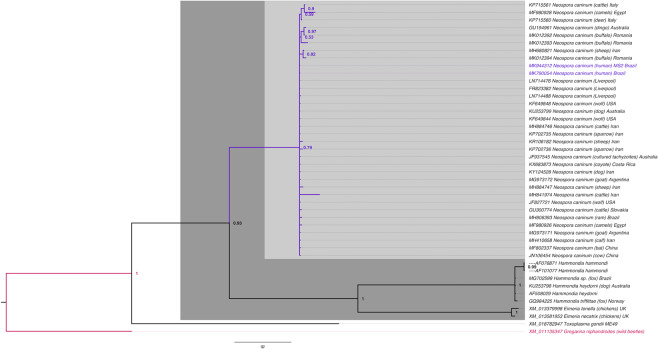
Phylogenetic tree of *Neospora caninum* (GenBank: MK790054; MK944312). Evolutionary history was inferred from the Bayesian Inference tree with the probability scores for the Nc5 gene. Bar, 0.2 changes per nucleotide position. The sample sequences obtained in this study are indicated in blue.

These two Nc5 gene samples were sent for sequencing, and both shared 100% similarity with each other and 100% homology for *N. caninum* Liverpool (GenBank: LN714476). The highest homology (98–99%) was obtained for *N. caninum* with strains from other countries (Fig. 1). ITS1 gene samples shared 100% homology for *N. caninum* Liverpool (GenBank: U16159) after sequencing.

For the B1 gene of *T. gondii*, 16 samples presented bands compatible with the positive control in the PCR results, with 5.5% positivity in cord blood and 2.5% positivity in placental tissue. Detailed information on PCR positive samples are presented in the Supplementary Table S1.

### Histopathological analysis

Histopathological analyses of 75 placenta samples (selected from among the samples showing PCR positivity and serological positive, prioritizing higher serological titles for both parasites) stained with hematoxylin-eosin were performed. These samples showed no structures characteristic of tissue cysts or inflammatory infiltrate.

## Discussion

Changes in the maternal immune status occur during pregnancy to maintain fetal survival, and this immunosuppression may leave pregnant woman more prone to infections^17,18^. Under healthy conditions, these infections are typically kept under control during pregnancy; however, the immature immune system of the fetus leaves it vulnerable to parasites that are able to cross the uteroplacental barrier^10^. The transplacental hematogenic route is the most common route of maternal-fetal parasite transmission^19^. *T. gondii* and other parasites in the phylum Apicomplexa actively penetrate their host cells *in vitro*, and this process is also possible *in vivo*^20^.

In the present study, 24.3% seropositivity for anti-*N. caninum* antibodies was found, suggesting human exposure to the parasite. The seropositivity rate was higher in the present study than the rate of 5% seropositivity found by Lobato *et al*.^13^, in 91 cord blood samples. Studies by Ibrahim *et al*.^14^, found a 7.92% (8/101) seroprevalence among pregnant women for *N. caninum*, Tranas *et al*.^15^, found 6.7% (69/1,029) seropositivity in blood bank samples, and Oshiro *et al*.^16^, found 26.1% (81/310) positivity in HIV-positive patients. The variations in the seropositivity rates found in several studies may be attributed to the study populations and the climatic and environmental factors of each region, as some authors have reported an association between climatic factors and risk factors for *N. caninum* infection in cattle^2,21,22^. The sporulation and survival of coccidial oocysts in the environment may be favored by temperature and humidity^2^.

Of all the samples tested, only 8.4% presented concomitant seropositivity to *T. gondii*. In the literature, there have been reports of seropositivity for both parasites: Ibrahim *et al*.^14^, reported 5.94% positivity, and Oshiro *et al*.^16^, reported 25.2% positivity. However, the extent of *N. caninum* and *T. gondii* coinfection in humans is still unknown. To decrease the possibility of cross reaction, in the present study was used a serological cut off point of 1:50 and only fluorescent reactions along the periphery of the parasite were considered positive.

In a study by Paré *et al*.^23^, complete peripheral fluorescence of the parasite was considered a positive response and apical fluorescence a negative response because the conservation of antigens in the apical organelles of a variety of Apicomplexa parasites may be responsible for cross-reactivity^24^. Dilutions equal to or greater than 1:50 in the IFAT may be considered appropriate to avoid cross-reactivity between *N. caninum* and *T. gondii* in serum samples from some hosts^13,25^.

IFAT positive samples and molecular biology for *N. caninum* demonstrated western blot reactivity for rNcSRS2 (Nc-p43) surface antigen which is immunodominant, highly immunogenic, well conserved^26^ and does not cross react with *T. gondii*^27^.

The two positive PCR samples for *N. caninum* were not IFAT positive, but showed weak reactivity by western blot, demonstrating that western blot can be used as a complementary serological method in the diagnosis of neosporosis. In the literature there are reports of positive PCR samples that tested negative in serology tests in studies carried out with dogs^28,29^, and in a study carried out with bovines, in tests with aborted fetal tissues, the mother tested negative for *N. caninum* by IFAT and ELISA and positive by PCR, with these samples showing poor reactivity on a western blot test^30^.

Therefore, explanations for this fact can be attributed to the inability of some individuals to synthesize detectable antibodies against *N. caninum* due to acquired or innate immunotolerance^30^, or also to the previous chronic infection with antibodies not detectable in the 1:50 dilution^28^. In studies carried out with mice, it has been demonstrated the appearance of IgM antibodies after 7 days of infection by *N. caninum* and the production of IgG antibodies after 14 days of infection^31^. This reinforces the possibility of the infection being acquired at the end of pregnancy with the mother still seronegative at delivery, as with *T. gondii* infections^32^.

Samples were considered positive when Nc5 and ITS1 were positive. Of the 201 cord blood samples and 201 placental tissue samples analyzed, two cord blood samples showed PCR positivity for *N. caninum* using primers for the Nc5 and ITS1 region, and these samples were negative for *T. gondii*. After sequencing for Nc5 gene (GenBank: MK790054; MK944312), the samples demonstrated 98%-100% identity with several strains in the database, and for ITS1 gene (GenBank: MN731361) shared 100% homology for *N. caninum* Liverpool, suggesting that these sequences really represented samples of *N. caninum*. The phylogenetic tree showed a cluster of *N. caninum* among samples from around the world and different hosts.

Nc5 sequences were used to construct the phylogenetic tree, because unlike ITS1, the Nc5 gene is highly specific and excludes other species from the Toxoplasmatinae subfamily^33^, which strengthens the molecular diagnosis of the present study.

The positivity found for the Nc5 and ITS1 genes corroborate literature data. The use of nested-PCR methods directed to the Nc5 and ITS1 genes to detect *N. caninum* DNA may increase sensitivity and detection rate^34–36^.

The present study found positive molecular biology results for two umbilical cord blood samples but not for the corresponding placental samples. Because this is the first report of *N. caninum* in human samples, further studies are needed to clarify these findings. In studies with cows experimentally inoculated at different stages of pregnancy, some authors have reported that histopathological changes are less frequent at more advanced stages of pregnancy, suggesting that gestational age influences the outcome of placental infection^37,38^.

In an experimental study conducted by Ho *et al*.^39^, with pregnant monkeys (*Macaca mulatta*), the sporadic and inconsistent distribution of *N. caninum* in tissues other than those from the central nervous system was proposed to be a manifestation of constant dissemination of a small number of parasites into the bloodstream.

Human neosporosis is still an uncertain issue, despite serological evidence of human exposure, primarily in immunocompromised populations^10,13,15^. Considering the high efficiency and prevalence of vertical transmission of *N. caninum* in cattle^40^ and its close relationship with *T. gondii*, the possibility of Neospora posing a risk to human pregnancy should not be ruled out. Experimental studies with nonhuman primates indicated susceptibility to transplacental infection, and fetal lesions caused by *N. caninum* infection were similar to those induced by *T. gondii* infection^41^. An *in vitro* study has shown that human trophoblasts and cervical cells are readily infected by *N. caninum*, although they show differences in susceptibility to infection, cytokine production and cell viability^42^.

In this study, a significant association between seropositivity for *N. caninum* and the presence of animals as well as the presence of dogs was observed. Canids are known to be the definitive, exclusive hosts of *N. caninum*^2^. Some authors have reported that the presence of dogs on rural properties may be related to an increased likelihood of infection in cattle, thus highlighting the role of dogs in the epidemiological chain of neosporosis in farm animals^43–45^. Since dogs are definitive hosts and excrete oocysts in feces, the potential for human exposure to *N. caninum* is high^14^. The presence of dogs may be related to *N. caninum* seropositivity in the analyzed pregnant women, but additional studies in this area are necessary to establish this correlation.

In the present study, 26.8% seropositivity for anti-*T. gondii* IgG antibodies was observed. Being 73.1% were seronegative for the presence of IgG antibodies, and 100% were negative for IgM. According to Villard *et al*.^46^, the presence of specific IgG and the absence of IgM antibodies are indications of previous infection, however, the infection can be acquired at the end of gestation, with the mother still being seronegative at birth^32^. The prevalence of IgG antibodies among pregnant women in Brazil is variable, and it can reach 63.03%^47–49^.

A significant association was found between *T. gondii* seropositivity and basic sanitation (access to sewage and treated water) with p = 0.04, but no significant associations were found with other risk factors. According to Silva *et al*.^50^, a lack of basic sanitation is associated with risk factors for *T. gondii* infection, with low socioeconomic level, low educational level, older age, soil management and contact with cats being considered more important risk factors in pregnant women in Brazil^6^. In the analyzed samples, there was no significant correlation between older age and serological positivity, perhaps because the pregnant women composing the study group were young.

The use of PCR analysis in the determination of intrauterine *T. gondii* infection allows early diagnosis and avoids the use of invasive procedures for the fetus^51^. In this study, we observed 5.5% positivity in cord blood and 2.5% positivity in placental tissue for the B1 gene, even with the exclusion of acute infection confirmed by serology. Postnatal screening may be associated with the detection of these parasites in amniotic fluid, the placenta and cord or neonate serum and may be a management strategy complementary to prenatal diagnosis^46^.

The B1 gene has approximately 35 copies and is highly conserved in all strains^52^. According to Jones *et al*.^53^, primers for the B1 gene have higher specificity because they do not amplify DNA from a variety of bacterial and fungal species and because, even in the presence of increasing amounts of human DNA, the sensitivity of the reaction remains unchanged; it is able to detect 50 femtograms (corresponding to a single organism) of *T. gondii* DNA.

In conclusion, the seroprevalence of *N. caninum* can be indicative of parasite exposure, and the presence of dogs may be associated with seropositivity. Additional studies are needed to clarify possible risk factors related to *N. caninum*. The PCR DNA detection results indicate that the role of *N. caninum* in human pregnancy still needs to be elucidated in order to determine the extent and importance of human exposure, given that the parasite has thus far not been isolated from human tissues. These findings may contribute to implementation of diagnostic tests in routine prenatal screening. The seroprevalence for *T. gondii* in pregnant women found in the present study was low compared with that found in other regions of Brazil, and lack of basic sanitation represented an important risk factor. However, seronegativity may indicate susceptibility to infection.

## Methods

### Ethics statement

The study was approved by the Ethics Committee for Research Involving Human Beings of the Federal University of Mato Grosso do Sul (UFMS) on 03 November 2016, document number 1.804.047. All included patients accepted the conditions of the study and signed the free informed consent form. All methods were carried out in accordance with relevant guidelines and regulations.

### Sample collection

This study is an analytical cross-sectional study. Between January and May 2017, a total of 201 cord blood and placental tissue samples were collected from pregnant women admitted to the delivery room and surgical center of Cândido Mariano Maternity Hospital, located in Campo Grande, Mato Grosso do Sul, Brazil.

Immediately after delivery, umbilical cord blood was collected in a vacutainer tube containing K_2_ EDTA for molecular analysis and a clot activator tube for serological analysis. Placental fragments weighing 1–2 grams were collected from the fetal (or chorionic) and maternal ends of the placental hilus for molecular and histological analyses^54^.

Data were collected from the patients’ charts and from a form completed by the patients that evaluated the following variables: age, gestational age, number of prenatal consultations, problems in previous pregnancies, and living conditions and habits (consumption of raw or undercooked meat; work or leisure activities involving soil; domestic animal raising; presence of cats and/or dogs in the home; and presence of basic sanitation/access to sewage collection or treated water).

The patients included in the study were healthy pregnant women with a normal pregnancy who were in initial labor and admitted to the same maternity sector.

### Serology

#### IFAT

The indirect fluorescent antibody test (IFAT) for the detection of anti-*N. caninum* antibodies was performed using an Imunoteste Neospora (RIFI) commercial diagnostic Kit (Imunodot diagnósticos, Jaboticabal-SP, Brazil) following the manufacturer’s recommendations with adaptations. Previously established positive and negative human serum controls provided by Oshiro *et al*., were used^16^. Samples were considered positive at a dilution of 1:50.

The IFAT for the detection of anti-*T. gondii* antibodies was performed using an Imuno-Con Toxoplasmose kit (WAMA Diagnóstica, São Carlos-SP, Brazil) following the manufacturer’s recommendations. Samples were considered positive at a dilution of 1:64.

For both serological tests of the 201 samples, human anti-IgG and anti-IgM fluorescence conjugate at 1:100 dilution (conjugated with fluorescein isothiocyanate; Sigma-Aldrich, St. Louis, Missouri, USA) were used. The slides were observed using a fluorescence-equipped microscope (Axioskop- Carl Zeiss, Germany) (epi-lighting system) with a 40× objective.

Fluorescent reactions along the periphery of the parasite were considered positive. In the negative reactions, the parasites on the slide did not show fluorescence, or the fluorescence was located at only one end, characterized as polar coloration or an apical reaction. Samples with peripheral fluorescence of total tachyzoites were considered positive^23^.

#### Western blot

*N. caninum* rNcSRS2 partial recombinant sequence (Nc-p43)^55^ protein was separated on 12% SDS-PAGE gel and transferred to PVDV membrane (GE Healthcare, UK) at 25 mA overnight (Supplementary Protocols S1).

### Molecular biology

#### DNA isolation

Approximately 300 microliters (µl) of cord blood from each sample (201 total) and 50 milligrams of placental tissue from each sample (201 total) were subjected to DNA extraction using a protocol adapted from Regitano and Coutinho^56^ (Supplementary Protocols S1).

Samples were quantified via spectrophotometry (NanoDrop ND-1000, Uniscience) and diluted to 100 nanograms for PCR. The viability of the samples and DNA quality were evaluated using primers for the human β-globin gene as described by Bauer *et al*.^57^.

#### PCR for *neospora caninum* and *toxoplasma gondii*

For detection of *N. caninum*, the primers NP21 and NP4 were used for the primary amplification and primers NP7 and NP4 were used in the secondary reactions to target the Nc5 gene as described by Yamage *et al*.^33^. Primers for internal transcribed spacer (ITS1) region was used out with four oligonucleotides as described by Buxton *et al*.^58–60^ (Supplementary Protocols S1).

For detection of *T. gondii*, was used primer to perform simple PCR targeting the repetitive and conserved B1 gene^61^, a nested PCR was also performed using N2-C2 primers, which amplified a 97-bp product of the B1 gene^62^ (Supplementary Protocols S1).

Negative (ultrapure water) and positive (*N. caninum* NC‐1 strain and *T. gondii* RH strain) controls were included with all PCR reactions. To increase the sensitivity of the assay, each DNA sample was tested in triplicate.

The final product was visualized on a 1.5% agarose gel stained with ethidium bromide (EtBr).

Samples yielding an expected PCR product size for *N. caninum* were purified using a PureLink quick gel extraction kit (Invitrogen, Carlsbad, CA) and DNA-sequenced at René Rachou Research Center (Oswaldo Cruz Foundation-FIOCRUZ) in an automatic sequencer (ABI Prism 3730XL Genetic Analyzer, Applied Biosystems, EUA) with a 48-capillary DNA analysis system.

#### Phylogenetic tree construction

Using the BLASTn program, sequences available from GenBank was aligned with the sequence of the Nc5 gene (GenBank: MK790054; MK944312). The Mega 6.0 program^63^ was used to align the sequences taken from GenBank and construct a database that contained all similar sequences obtained from the analysis. Using the MrBayes 3.2.6 program was performed a Bayesian phylogenetic analysis for the Nc5 gene and the results were plotted using the FigTree 1.4.2 program^64–66^.

The topology of the tree was used to generate a 50% majority rule consensus, with the percentage of samples recovering any particular clade representing the posterior probability of a clade (1 = 100%). No manual editing of the tree was performed. The *Gregarina niphandrodes* (GenBank: XM_011135347) dataset was used as the outgroup in the phylogenetic tree.

#### Histopathological analysis

Fragments of placental samples weighing 1 to 2 grams collected during delivery were immediately fixed in 10% buffered formalin for 24 hours, processed (xylol alcohol), embedded in paraffin, sliced into a final thickness of 5 µm and placed on a slide for hematoxylin-eosin staining. The slides were visualized at 400X magnification to examine placental morphology.

### Statistical analysis

The collected data were tabulated and analyzed using the statistical software IBM SPSS Statistics version 20 (Inc., Chicago, Illinois, USA). The χ^2^ test, Fisher’s exact test and odds ratios were used to assess associations between the variables (consumption of raw/undercooked meat, work or leisure activities involving soil, domestic animals, cat, dog, basic sanitation) and the serology results. p values less than 0.05 were considered statistically significant.

## Supplementary information

Supplementary Information.
