# Supplementary material for: Serological and molecular detection of Neospora caninum and Toxoplasma gondii in human umbilical cord blood and placental tissue samples
Source: Sci Rep. 2020 Jun 3;10:9043. doi: 10.1038/s41598-020-65991-1 (PMC7271125; doi:10.1038/s41598-020-65991-1)
Supplement: Supplementary file 1 — Supplementary Information. [file 41598_2020_65991_MOESM1_ESM.pdf]

Supplementary Information

Serological and molecular detection of *Neospora caninum* and *Toxoplasma gondii* in human umbilical cord blood and placental tissue samples

Pâmella Oliveira Duarte[1], Leandra Marla Oshiro[2], Namor Pinheiro Zimmermann[3], Bárbara Guimarães Csordas[4], Doroty Mesquita Dourado[5], Jacqueline Cavalcante Barros[6], Renato Andreotti[6]*

[1]Programa de Pós-Graduação em Doenças Infecciosas e Parasitárias-Universidade Federal de Mato Grosso do Sul (UFMS), Campo Grande-MS, Brasil; [2]Laboratório de Biologia Molecular do Carrapato, Departamento de Sanidade Animal, Embrapa Gado de Corte, Campo Grande-MS, Brasil; [3]Professor do Curso de Medicina Veterinária do Centro Universitário da Grande Dourados-UNIGRAN, Brasil; [4]Bolsista de Pós-Doutorado- FUNDAPAM, Laboratório de Biologia Molecular do Carrapato, Departamento de Sanidade Animal, Embrapa Gado de Corte, Campo Grande-MS, Brasil, [5]Laboratório de Toxonologia e Plantas Medicinais-Uniderp Agrárias, Brasil; [6]Empresa Brasileira de Pesquisa Agropecuária, Embrapa Gado de Corte, Campo Grande-MS, Brasil.

*Correspondence: renato.andreotti@embrapa.br

Supplementary Protocols S1:

Serology

Western blot

N. caninum Nc-p43 surface protein was separated on 12% SDS-PAGE gel and transferred to PVDV membrane (GE Healthcare, UK) at 25 mA overnight. The membrane was blocked in 5% skim powdered milk for 1 hour, washed in phosphate buffered saline/Tween (PBST) and incubated with positive and negative human serum (1:50 + PBST) for 1 hour in shaker at 37°C and washed again with PBST. The membrane was incubated anti-IgG antibodies conjugated with peroxidase (Sigma Chemicals, USA), diluted 1:1000 with PBS and incubated for 1 hour at 37°C in shaker. The membrane was washed with PBST and revealed (50 mM Tris-HCl pH 7.5; 0.3% nickel solution; 15 µl hydrogen peroxide; 0.006 mg 3,3'-diaminobenzidine substrate).

Molecular biology

DNA isolation

Approximately 300 microliters (µl) of cord blood from each sample (201 samples total) was added to an Eppendorf tube containing 2 µl of proteinase K (20 mg/ml) and 500 µl of sodium dodecyl sulfate (SDS) 20%. The samples were homogenized and incubated at 65°C for 1 hour. After incubation, 800 µl of chloroform was added and the samples were vortexed, followed by the addition of 350 µl of protein precipitation solution (6 ml of potassium acetate, 1.1 ml of glacial acetic acid, and 2.9 ml of water). The samples were centrifuged at 18,000 x g for 10 minutes, and the aqueous phase was transferred to a new tube containing 1 ml of absolute ethanol, which was inverted until precipitate formation. After centrifugation at 13,000 rpm for 5 minutes, the supernatant was discarded, 1 ml of 70% ethanol was added, and the sample was centrifuged again at 13,000 rpm for 2 minutes. The supernatant was then discarded, and the pellet was dried at 37°C for 15 minutes. Next, the DNA was hydrated in 50 µl of ultrapure water and incubated at 37°C for 1 hour in a thermoblock.

Approximately 50 milligrams of placental tissue from each sample (201 samples total) was macerated in 800 µl of BL lysis buffer (100 mM TRIS-HCl; 10 mM Ethylenediamine tetraacetic acid/ EDTA, pH 7.6-8.0 and 300 mM NaCl). After homogenization, 160 µl of 10% SDS preheated to 55°C and 20 µl of proteinase K (20 mg/ml) were added, and the samples were incubated overnight in a 55°C water bath. After the incubation period, the samples were centrifuged at 6,000 x g for 5 minutes, and 800 µl of the supernatant was transferred to a new tube containing 300 µl of NaCl (6 M). The samples were vortexed for 10 seconds and centrifuged at maximum speed for 30 minutes at 4°C. After, the supernatant was transferred to a new tube containing 900 µl of isopropanol and incubated at -20°C for 2 hours. After centrifugation at 10,000 x g for 10 minutes, the supernatant was discarded, and 1 ml of 70% ethanol was added. After another centrifugation at 10,000 x g for 5 minutes, the supernatant was discarded again, and the pellet was dried at 37°C for 15 minutes. The DNA was hydrated in 100 µl of ultrapure water for incubation at 55°C in a thermoblock for 40 minutes.

PCR for ***Neospora caninum*** **and** ***Toxoplasma gondii***

Reactions were prepared with a final volume of 25 µl and contained the following components: 1X PCR buffer (200 mM Tris-HCl pH 8.4 and 500 mM KCl); 1.5 mM potassium chloride; 0.2 mM dNTP mix (Invitrogen™), 0.2 µM of each primer and 1 unit of Platinum® Taq DNA Polymerase (Invitrogen™). The reactions were conducted on a T100 thermal cycler (Bio Rad™) and subjected to the following program for *N. caninum* (Nc5 gene): five minutes for initial denaturation at 94°C, followed by 40 cycles of one minute at 94°C, one minute at 62°C and three minutes at 72°C, and ending with five minutes at 72°C for final extension; (ITS1 gene) five minutes for initial denaturation at 95°C, followed by 35 cycles of one minute at 94°C, one minute at 48°C and one minute at 72°C, and ending with five minutes at 72°C for final extension.

The following program was used for *T. gondii (*B1 gene): five minutes for initial denaturation at 94°C, followed by 40 cycles of one minute at 94°C, one minute at 60.7°C and two minutes at 72°C, and ending with five minutes at 72°C for final extension. A nested PCR was also performed repeated under the same

conditions as described above. The final product was visualized on a 1.5% agarose gel stained with ethidium bromide (EtBr).

Supplementary Figure S1

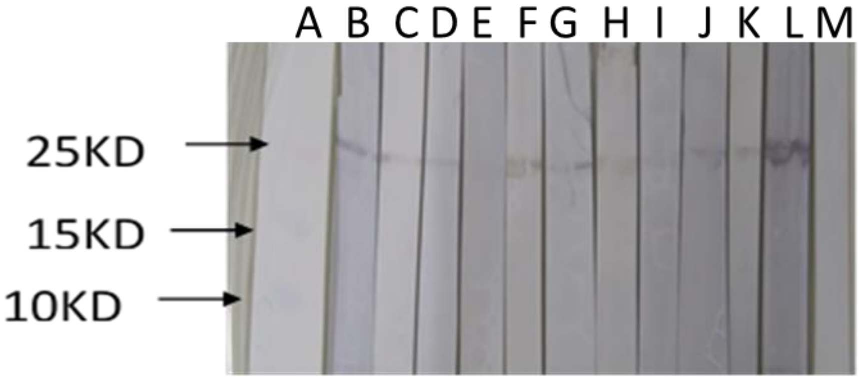

Supplementary Figure S1: Western blotting Nc-p43 containing the recombinant protein fragment with a 29KDa. (A) Molecular weights of markers; (B-L) anti-human IgG peroxidase-conjugated antibodies from human serum positive for *N. caninum*; (M) Western blotting antibodies from human serum negative for *N. caninum.* The figure shown is representative of data from only one experiment performed individually for each serum sample.

Supplementary Table S1: Detailed information on PCR positive samples.

Samples	PCR (*N. caninum*)	IFAT (*N. caninum*)	Western blot	PCR (*T. gondii*)	IFAT (*T. gondii*)	Age	Consumption of raw/undercooked meat	Work or leisure activities involving soil	Domestic animals	Cat	Dog	Basic sanitation
47	Positive (cord blood)	Negative	Positive	Negative	_	28	No	Yes	No	No	No	Yes
50	Positive (cord blood)	Negative	Positive	Negative	_	31	No	No	Yes	Yes	Yes	No
4	Negative	_	_	Positive (placenta)	Negative	26	No	No	Yes	No	Yes	No
10	Negative	_	_	Positive (cord blood)	Positive	28	Yes	No	No	No	No	No
12	Negative	_	_	Positive (cord blood)	Negative	22	No	No	Yes	Yes	Yes	Yes
25	Negative	_	_	Positive (cord blood)	Positive	34	Yes	No	No	No	No	Yes
32	Negative	_	_	Positive (cord blood)	Negative	36	No	No	No	No	No	Yes
71	Negative	_	_	Positive (placenta)	Positive	32	No	No	Yes	No	Yes	No
86	Negative	_	_	Positive (cord blood)	Negative	20	No	No	Yes	No	Yes	Yes
92	Negative	_	_	Positive (placenta)	Negative	29	No	No	No	No	No	No

115	Negative	_	_	Positive (placenta)	Positive	24	No	No	No	No	No	Yes
127	Negative	_	_	Positive (cord blood)	Negative	31	Yes	No	Yes	No	Yes	Yes
135	Negative	_	_	Positive (cord blood)	Positive	20	No	No	Yes	No	Yes	Yes
140	Negative	_	_	Positive (cord blood)	Negative	25	No	No	Yes	No	Yes	Yes
142	Negative	_	_	Positive (placenta)	Negative	33	No	No	No	No	No	Yes
147	Negative	_	_	Positive (cord blood)	Negative	26	No	No	No	No	No	Yes
162	Negative	_	_	Positive (cord blood)	Negative	26	No	No	Yes	No	Yes	Yes
177	Negative	_	_	Positive (cord blood)	Negative	24	No	Yes	Yes	Yes	Yes	Yes

Note: Samples 4, 47 and 177 have had a previous pregnancy abortion.
